# MicroRNA biomarkers of type 2 diabetes: A protocol for corroborating evidence by computational genomics and meta-analyses

**DOI:** 10.1371/journal.pone.0247556

**Published:** 2021-04-06

**Authors:** Hongmei Zhu, Siu-wai Leung

**Affiliations:** 1 State Key Laboratory of Quality Research in Chinese Medicine, Institute of Chinese Medical Sciences, University of Macau, Macao, China; 2 Shenzhen Institute of Artificial Intelligence and Robotics for Society, Shenzhen, China; 3 Edinburgh Bayes Centre for AI Research in Shenzhen, College of Science and Engineering, University of Edinburgh, Scotland, United Kingdom; University of California San Francisco, UNITED STATES

## Abstract

**Background:**

Few microRNAs were found consistently dysregulated in type 2 diabetes (T2D) that would gain confidence from Big Pharma to develop diagnostic or therapeutic biomarkers. This study aimed to corroborate evidence from eligible microRNAs-T2D association studies according to stringent quality criteria covering both biological and statistical significance in T2D for biomarker development.

**Methods and analyses:**

Controlled microRNA expression profiling studies on human with T2D will be retrieved from PubMed, ScienceDirect, and Embase for selecting the statistically significant microRNAs according to pre-specified search strategies and inclusion criteria. Multiple meta-analyses with restricted maximum-likelihood estimation and empirical Bayes estimation under the random-effects model will be conducted by *metafor* package in R. Subgroup and sensitivity analyses further examine the microRNA candidates for their disease specificity, tissue specificity, blood fraction specificity, and statistical robustness of evidence. Biologically relevant microRNAs will then be selected through genomic database corroboration. Their association with T2D is further measured by area under the curve (AUC) of receive operating characteristic (ROC). Meta-analysis of AUC of potential biomarkers will also be conducted. Enrichment analysis on potential microRNA biomarkers and their target genes will be performed by iPathwayGuide and *clusterProfiler*, respectively. The corresponding reporting guidelines will be used to assess the quality of included studies according to their profiling methods (microarray, RT-PCR, and RNA-Seq).

**Ethics and dissemination:**

No ethics approval is required since this study does not include identifiable personal patient data.

**Protocol registration number:**

CRD42017081659.

## Introduction

Type 2 diabetes (T2D) is characterized by insulin resistance and beta-cell dysfunction [1], which severity would progress gradually (or alleviate temporarily) for decades. Without appropriate and timely interventions, preferably in pre-diabetes (Pre-T2D) and early T2D, malfunctions would occur in multiple organs, especially the heart, blood vessels, eyes, kidneys, and nerves [2]. The International Diabetes Federation (IDF) estimated that 50% of the adult diabetics were still undiagnosed in 2017 and detectable Pre-T2D are still too late in disease states to manage [3]. Therefore, the demands for even earlier diagnosis of diabetes using molecular medicine are imminent. MicroRNAs represent a kind of promising biomarkers for early diagnosis and monitoring of complex diseases [4, 5] including T2D [6]. Recent studies found that the use of biomarkers in patient-selection exhibited higher overall success probabilities, compared to trials without biomarkers during the drug development [7, 8]. In addition, there have been patent applications regarding the use of microRNAs as disease-specific biomarkers (https://patentscope.wipo.int/search/en/result.jsf), such as (https://patentscope.wipo.int/search/en/detail.jsf?docId=WO2018125019&recNum=3&office=&queryString=FP%3A%28microRNA%29&prevFilter=&sortOption=Pub+Date+Desc&maxRec=3482).

MicroRNAs are small (approximately 22 nucleotides), endogenous, noncoding, highly stable, and gene-regulating RNAs. Their dysregulation were linked to many diseases [9, 10], including T2D [6, 11]. According to our pilot study [12], the controlled profiling studies on microRNAs in T2D were heterogeneous and inconsistent with one another; thus, they are subject to evaluation by proper meta-analysis which can integrate statistical evidence from multiple studies, estimates overall effect sizes, and assesses the reliability, credibility, and uncertainty of the evidence [13, 14]. Meta-analysis methods have been increasingly used in recent years for evidence-based clinical, biomedical, and social research [15, 16]. Since most of genomic studies have small sample sizes [17], meta-analysis would increase statistical power by combining multiple studies for identification of differentially expressed genes, biological networks and predictive models of diseases [17]. Our demonstrative study published in early 2015 tested use a proper statistical method, replacing the obsolete vote-counting method, for conducting non-trivial microRNA meta-analyses [12]. Since then, over 100 studies have cited and/or followed our pilot method for studying many other diseases. In the meantime, the present protocol will have been conducting to take stock of all microRNAs that are reliably associated with T2D, representing the largest meta-analytical cross-identification and cross-validation on microRNAs in T2D. According to our PubMed searches, the number of microRNA studies on T2D dramatically increased from 253 to 1404 (i.e. 5.5 folds) between 11 March 2014 and 3 April 2020. And eight systematic reviews [18–25] with various flaws published since our pilot study were found among the 1404 studies. There is a study [18] conducted a qualitative analysis without quality assessment and statistical meta-analysis. A study [19] followed our pilot method but it focuses on a single microRNA, namely miR-146a. Another study [20] replicated the methods and argumentation of our pilot study^12^. Assmann et al. [21] performed a qualitative analysis and Zhang et al. [24] conducted quantitative analysis on type 1 diabetes (T1D) rather than T2D. Gholaminejad et al. [22] and Zhou et al. [25] adopted an vote-counting method, which was abandoned by meta-analysts [14], to analyze diabetic nephropathy and retinopathy, respectively. Park et al. [23] focused on mixed data from both T1D and T2D nephropathy. Thus, an up-to-date and comprehensive meta-analysis since our publication in 2015 [12] is still missing to fill the gap to confirm which microRNAs are reliably associated with T2D (Table 1). On the other hand, deeper biological relevance should be evaluated after confirming statistical significance and before any large-scale population-based studies in order to establish a genetic testing panel of microRNAs specific for early T2D diagnosis. Those population-based studies that should recruit >10K participants and might cost millions of US dollars would not be justified in terms of cost-effectiveness without a deep meta-analysis to corroborate and cross-validate evidence in terms of both statistical significance and biological relevance of specific microRNAs. Therefore, the deep meta-analysis and genomic analysis as provided in the present study protocol is crucial to evidence-based selection of T2D-associated microRNAs for establishing a genomic testing panel.

**Table 1:** 

Study	Disease	microRNA	Meta-analysis	Subgroup and sensitivity analyses	Quality assessment	Other analysis	No of studies included
Our pilot study [12]	T2DM	***	✓	✓	✓	✗	38
Villard 2015 [18]	Obesity & T2D	**	✗	✗	✗	➀	26
Alipoor 2017 [19]	T2D	*	✓	✓	✓ (no results)	➁	10
Liang 2018 [20]	T2D	***	✓	✓	✓	✗	39
Assmann 2018 [21]	T1D	***	✗	✗	✓	➀	33
Gholaminejad 2018 [22]	Diabetic nephropathy (T1D & T2D)	***	✓ (vote-counting)	✓	✗	➀	53
Park 2018 [23]	Diabetic nephropathy (T1D & T2D)	**	✓	✓	✓	➁	14
Zhang 2020 [24]	T1D	***	✓	✗	✓	✗	17
Zhou 2020 [25]	Diabetic retinopathy	***	✓ (vote-counting)	✗	✓	➀	8
Our protocol	T2D	***	✓	✓	✓	➀➁➂➃➄	≈150^#^

Summary of included systematic reviews on diabetes.

^➀^, enrichment analysis;

^➁^, publication bias;

^➂^, genomic data integration;

^➃^, ROC (receive operating characteristic);

^➄^, AUC (area under the curve).

***, unspecified, encompassed microRNAs from different tissue types or sample sources;

**, encompassed a kind of microRNAs (such as circulating microRNAs) or encompassed microRNAs from one tissue type;

*, a single microRNA;

^✗^, con-performed;

^✓^, performed;

^#^, estimation number of eligible studies according to the current study searching.

## Methods and material

### Design

Meta-analysis and computational genomics.

### Information sources

Databases including PubMed, ScienceDirect, and Embase will be searched for T2D microRNA expression profiling studies.

### Search strategies

Basic search terms include diabetes, microRNA, expression and “profil*” will be used to search title, abstract and/or keywords for selection of studies comparing T2D with controls. For example, PubMed will be searched by following strategy: (‘miRNA’, ‘diabetes’ and ‘expression’ in Title/Abstract) or (‘miRNA’, ‘diabetes’ and ‘profil*’ in Title/Abstract) or (‘microRNA’, ‘diabetes’ and ‘expression’ in Title/Abstract) or (‘microRNA’, ‘diabetes’ and ‘profil*’ in Title/Abstract). Search strategies for the selected databases were tested from Nov 2019 to Jan 2020.

### Eligibility criteria and study selection

**Fig 1:**
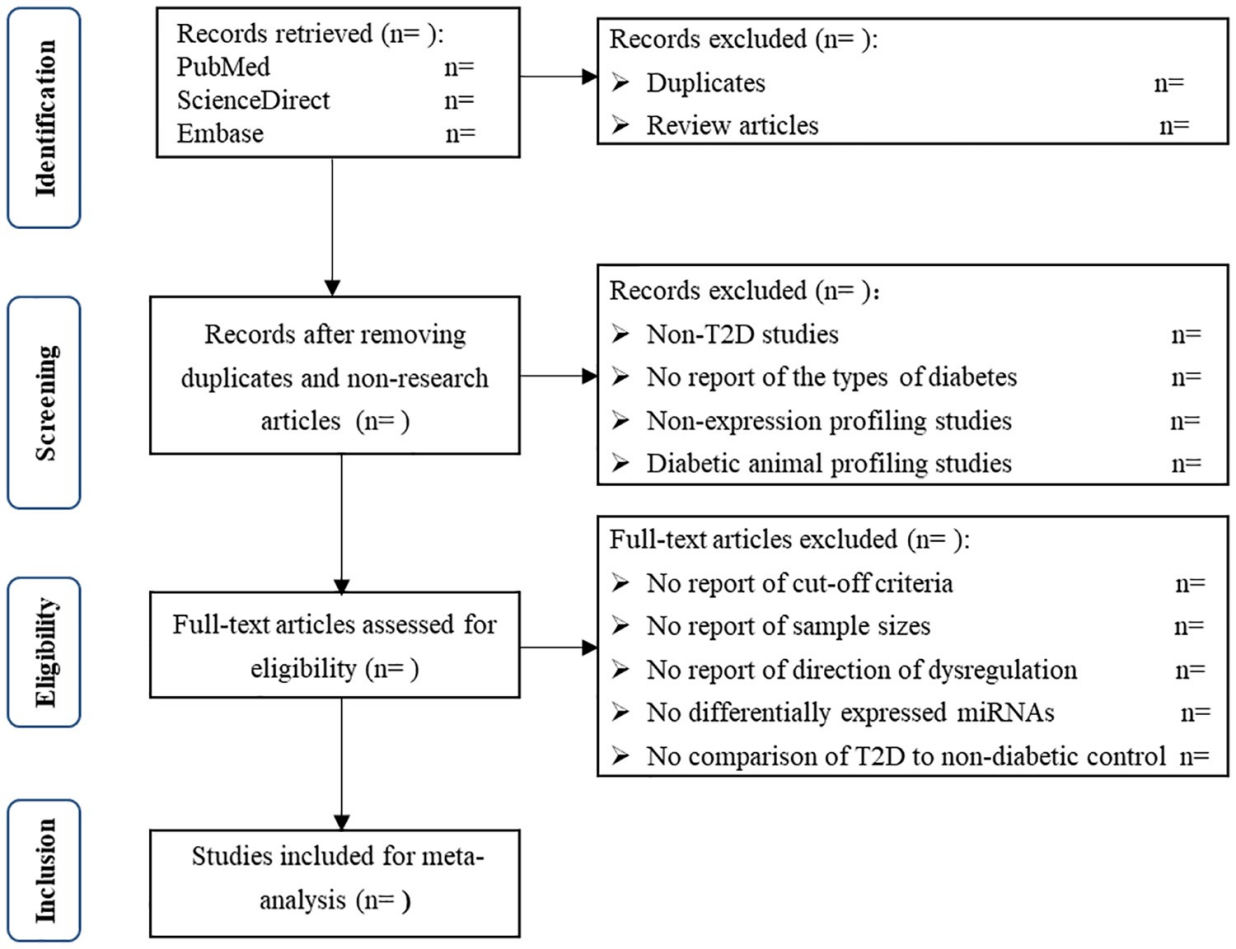
Flow diagram of study selection.

Eligible studies have to meet the inclusion criteria: (a) they are microRNA expression profiling studies on the participants with T2D; (b) they use diabetic and non-diabetic control samples for comparison; (c) they use microRNA expression arrays; (d) they report cut-off criteria of differentially expressed microRNAs and (e) they report sample sizes. The microRNA profiling studies using saliva or urine of the participants with T2D will be excluded, because we focus on microRNAs in blood and microRNAs in saliva and urine are mostly released from oral cancer [26] and urinary tract cancer [27–29], respectively. The PRISMA-compliant flow chart for study selection will be presented as shown in Fig 1.

### Data extraction and quality assessment

**Fig 2:**
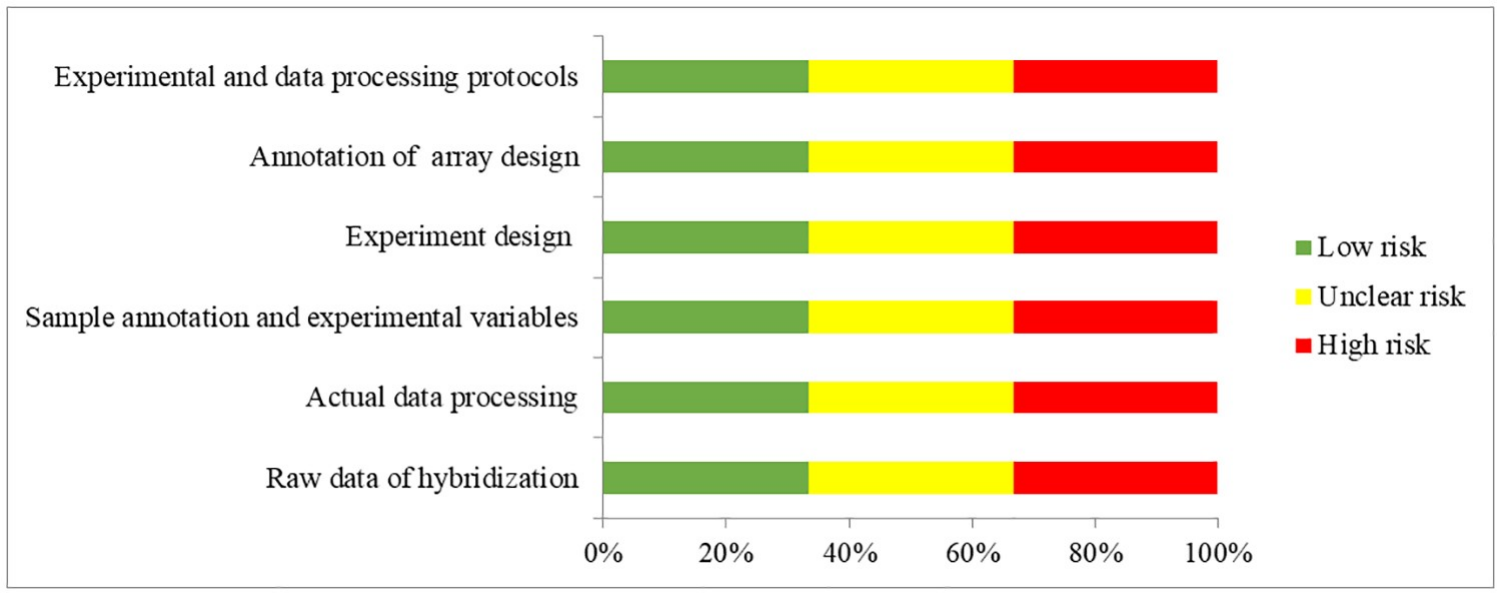
Quality assessment according to the MIAME guideline. Green bars, yellow bars and red bars respectively indicate the items were low risk, unclear risk and high risk.

**Fig 3:**
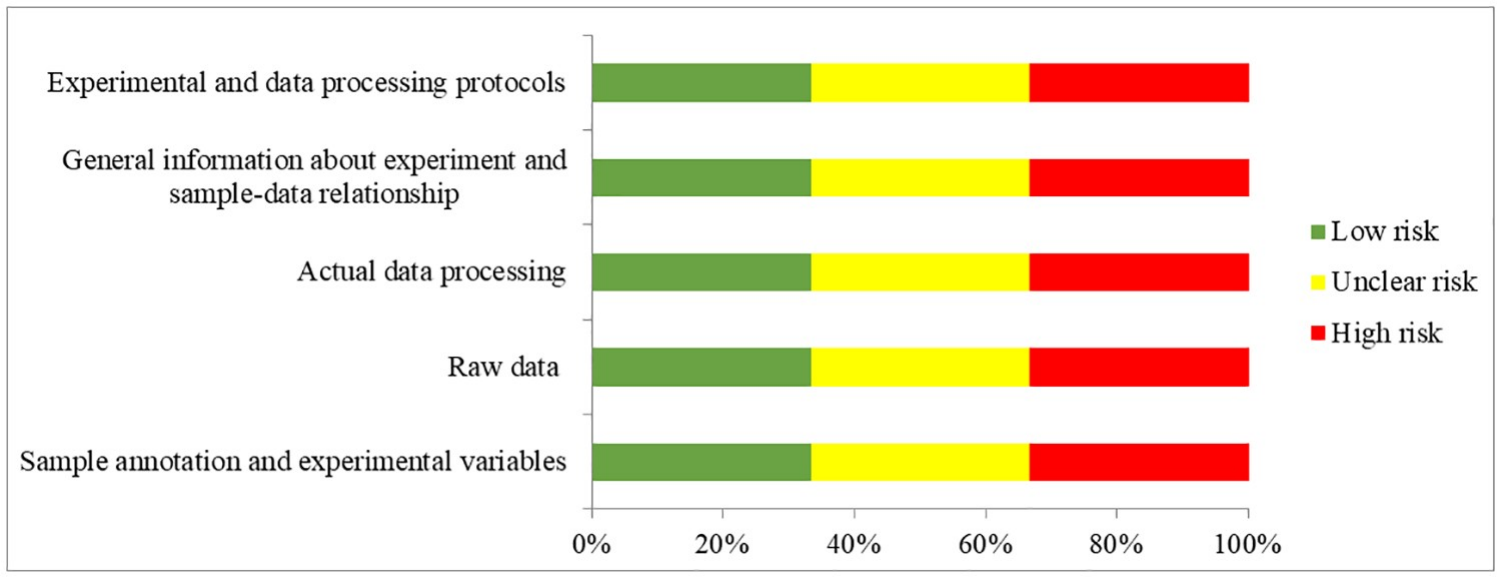
Quality assessment according to the MINSEQE guideline. Green bars, yellow bars and red bars respectively indicate the items were low risk, unclear risk and high risk.

From the full text and S1 Checklist of each expression profiling study, the following eligibility items will be collected and recorded: first author, year of publication, location of study, selection and characteristics of the participants with T2D, microRNA expression profiling platform, sample sizes, tissue types, cut-off criteria of up-regulated and down-regulated microRNAs and the list of differentially expressed microRNAs (Table 2). The extracted microRNAs will be alignment with miRBase version 22 [30] to unify the names before quality assessment. Quality assessment of microarrays will be performed according to the MIAME guideline version 2.0 [31]. Studies adopted qPCR-based microRNA arrays will be assessed according to the MIQE guideline [32] which should be corresponding to the MIAME guideline (Table 3 and Fig 2). RNA-Seq studies would be assessed according to MINSEQE guideline (Table 4 and Fig 3), proposed by FGED Society in 2012 (http://fged.org/projects/minseqe/). And the quality of transcriptomic parts of included studies will be assessed, including the collection of raw data, actual data processing, sample annotation, experimental design, array annotation, and data processing protocol. Each item is evaluated with low risk, unclear risk and high risk, suggesting high reproducibility, ambiguous reproducibility and low reproducibility, respectively.

**Table 2:** 

Study	T2D				Differentially expressed microRNAs				
	Country	Tissue	Clinical status	No. of samples (T2D/nondiabetic)	Platform	Cut-off criteria	Total	Upregulated	Downregulated
Study 1									
Study 2									
Study 3									
…									

Characteristics of human microRNA expression profiling studies (T2D vs nondiabetic controls).

**Table 3:** 

Source of bias	Raw data of hybridization	Actual data processing	Sample annotation and experimental variables	Experiment design	Annotation of array design	Experimental and data processing protocols
Study 1						
Study 2						
Study 3						
…						

Quality assessment according to the MIAME and MIQE guideline (for microarray and PCR study).

Each item of an included study is evaluated at low risk, unclear risk and high risk of bias.

**Table 4:** 

Source of bias	Sample annotation and experimental variables	Raw data	Actual data processing	General information about experiment and sample-data relationship	Experimental and data processing protocols
Study 1					
Study 2					
Study 3					
…					

Quality assessment according to the MINSEQE guideline (for RNA-Seq study).

Each item of an included study is evaluated at low risk, unclear risk and high risk of bias.

### Meta-analysis

Extracted data will be transferred to R with the *metafor* package [33] for meta-analysis under random-effects model. Both restricted maximum-likelihood estimation (REML) and empirical Bayes estimation (EB) will be employed to estimate the outcomes. The outcomes of differential expressed microRNA are presented as log_e_ (odds ratios) (LogOR), based on the numbers of dysregulation events in both the participants with T2D and non-diabetic control samples, with their 95% confidence intervals (CI). Adjusted *p* values less than 0.05 were considered statistically significant after Bonferroni corrections. The microRNAs statistically identified by both estimation methods (REML and EB) will be considered significant as differentially expressed in this meta-analysis. When compared to non-diabetic control groups, a significant logOR (> 0) indicates microRNA up-regulation. When compared to diabetic group, a significant logOR (> 0) indicates microRNA down-regulation. Differentially expressed microRNAs in the participants with T2D and non-diabetic control samples will be ranked according to the following order of importance (a) *p* values; (b) the number of consistent reports, and (c) OR values. Area under the curve (AUC) of receive operating characteristic (ROC) for each potential biomarker will be further preprocessed by *pROC* package [34] among studies with raw data on blood and blood fractions, before meta-analysis of AUC.

### Subgroup analysis

MicroRNAs are differentially expressed among complications or risk factors of T2D, tissue types and blood fractions, with corresponding overall effects and heterogeneities. Subgroup analyses will split the extracted data according to the complications/risk factors of T2D (heart failure, diabetic nephropathy, diabetic foot, hypertension, obesity, etc.), tissue types (blood, muscle, pancreas, liver, etc.) and blood fractions [serum, plasma, peripheral blood mononuclear cells (PBMCs), etc.] to compare microRNA expression profiles among complications/risk factors of T2D, tissue types and blood fractions (i.e. disease specificity, tissue specificity and blood fraction specificity). When examining tissue specificity, studies using serum, plasma, PBMCs will be classified as blood as they are from blood and aimed to investigate circulating microRNAs. And studies that do not report the pancreatic tissue is whole pancreas or pancreatic islets will be pooled with the other studies on pancreatic islets.

### Sensitivity analysis

Sensitivity analysis will performed on the sample size to test the robustness of findings. Meta-analyses will be repeated after excluding the studies whose sample sizes are less than 25 and 50, respectively. Laterza et al. [35] and Kosaka et al. [36] have demonstrated how circulating microRNAs may indicate the physiological state at tissue level. The microRNAs that circulate in the blood are in a stable form and remain stable even after multiple freeze-thaw cycles. They can be detected by least invasive techniques [37] and are specific to tissue and disease states [38]. If these microRNAs in circulating blood can serve as biomarkers, they would provide a less invasive approach to diagnosing and monitoring T2D. Therefore, the potential circulating biomarker candidates should be statistically significant and robustly up/down-regulated in sensitivity analyses (groups of sample sizes ≥ 50) and detectable in blood or blood fractions.

### Publication bias

Funnel plots will be generated to visualize possible publication bias. Begg test [39] and Egger test [40] using the package *metafor* will be performed to evaluate the statistical significance of the publication bias. For selecting microRNAs as biomarkers, we only consider the microRNAs with statistical significant effect sizes that are corrected by the trim-and-fill method.

### Genomic data integration

**Fig 4:**
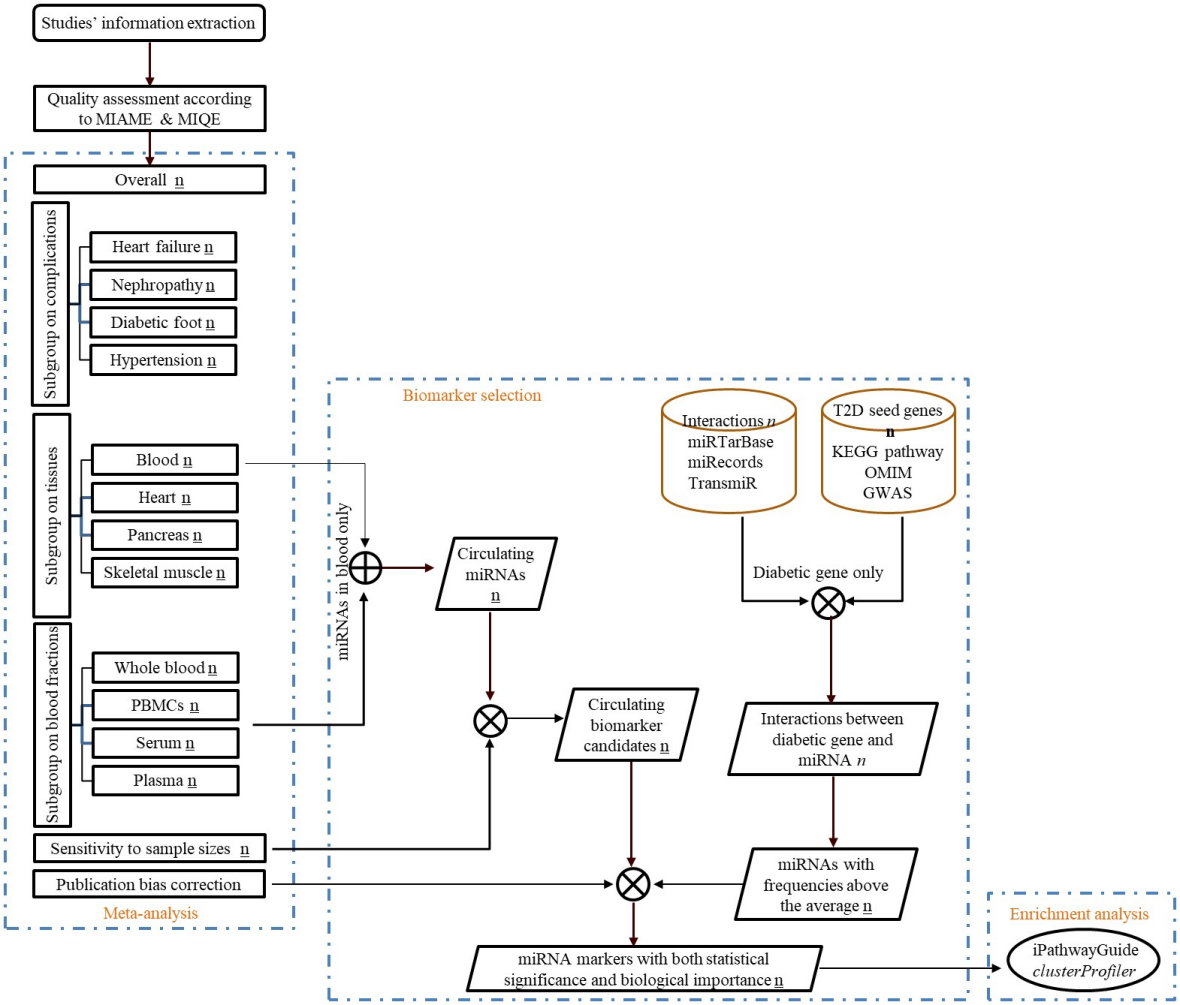
Flow diagram showing the overall design of this study and stepwise selection of circulating microRNA biomarkers. Statistical significant circulating microRNAs will be first identified by meta-analysis, which is filtering by sensitivity analysis to find the robust one. Experiment validated microRNA targets are used to further screen biologically significant microRNA markers according to well-established T2D genes. Number with underline, in italic and in bold indicates the number of statistically significant microRNAs, the number of interactions between microRNAs and targets, and the number of seed genes of T2D, respectivily. ⊕, the union of microRNAs. ⦻, the intersection of microRNAs.

A set of known genes closely associated with a disease are called seed genes. Seed genes of T2D will be retrieved from KEGG (Kyoto Encyclopedia of Genes and Genomes) database [41], OMIM (Online Mendelian Inheritance in Man) database [42] and GWAS (Genome-Wide Association Studies) Catalog (URL: https://www.ebi.ac.uk/gwas/; selection criteria: with a significance cutoff of *p*-value no more than 1*10^−10^). Interactions among human microRNAs and their experimentally validated target genes will be downloaded from the databases miRTarBase 7.0 [43], miRecords 4.0 [44], and TransmiR 2.0 [45]. MicroRNA names will be aligned with miRBase version 22 [30]. Conversion of target gene symbols to gene IDs will be done by bioDBnet (biological DataBase network) [46] since a single gene may have multiple symbols but one unique ID. T2D seed genes will be used as a filter to identify corresponding microRNAs associated with T2D from above microRNA-target gene interactions. The frequencies of microRNAs, which represent the strength of the association between the microRNA and T2D, will be calculated after the identification. A high frequency indicates that a microRNA would interact with more seed genes or there are more articles reporting such interactions between the microRNA and seed genes. The microRNAs with above-average frequencies will be grouped for category intersection and visualized in a Venn diagram to identify potential circulating biomarker, such that our potential microRNA biomarkers would have both statistical significance and biological importance. The overall design of this protocol and selection of potential biomarkers will be shown in a flow chart (Fig 4). Enrichment analysis will be further performed on selected potential microRNA biomarkers and their target gens (both seed and non-seed genes) using iPathwayGuide (https://www.advaitabio.com/ipathwayguide) and *clusterProfiler* package [47] for R, respectively to validate whether those microRNAs are enriched in pathways involved in T2D.

## Publication plan

The protocol of this study has been registered in PROSPERO (International Prospective Register of Systematic Reviews) following the PRISMA-P guideline (S1 Checklist) with the registration number CRD42017081659. The meta-analyses and genomic integration will be conducted accordingly, and the final report of the corroborated evidence will be submitted to a peer-reviewed journal for publication.

## Supporting information

S1 Checklist The PRISMA-P checklist. (PDF)
